# Uric Acid Solvents in Gout and Gravel

**Published:** 1893-11-18

**Authors:** 


### New Drugs and Preparations.

### URIC ACID SOLVENTS IN GOUT AND GRAVEL.

The labours of Sir William Roberts have marked out a wide difference in the means to be employed in combating the various effects of uric acid. At first sight it would seem rational to seek to prevent the deposit of uric acid in the tissues, the kidneys, or the bladder by some one substance which has known solvent powers. It is easy to take any number of reagents and put a crystal of uric acid in each, and note the power they have of dissolving it. But inside the body all the conditions are altered. There is an ever-changing medium —the blood and lymph, "a living stream, with high powers of self-adjustment," which interferes with the experiment. It would seem impossible to maintain the entire stream in a condition favourable to the process we desire to carry on. If we charge it with alkalies these are quickly swept away; and, moreover, what we generally desire to do is something quite different to the solution we attempt in our test tubes. We desire to *prevent* the deposition of uric acid, otherwise the quadriurate is converted in the gouty patient into a hydrated biurate, this is changed into the anhydrous biurate, and the latter finally precipitated as crystals. The rapidity of the process, or the reverse, depends on the amount of urates present, and also on that of the sodium salts, while none of the ordinary remedies for gout would seem to have any power to retain the uric acid in a soluble form. If the precipitation has taken place—and it first occurs in the synovial fluids and the cartilages where there is most sodium salt—and if the percentage of urates in the blood serum afterwards becomes very low, the crystals may in time again dissolve. This happens normally when from any cause the state of the patient changes, and he no longer produces urates in excess of the amount which the kidneys can excrete. Thus we sometimes see, under careful diet, the attacks become rarer, and the tophi and concretions about the joints gradually absorbed in the course of time. According to this view of gout, we must separate the lithæmic condition from uratosis, the precipitation of the uric acid compounds. The whole of the pain and injury seen in an acute attack are due, not to any poisonous action of uric acid, but simply to the mechanical irritation and obstruction produced by crystallisation. Now it has been the custom to employ a certain class of drugs to prevent this deposit. The alkalies, lithia, and of late piperazine and the salicylates have a reputation of this kind as tending to prevent uratosis, but Sir William Roberts failed to find that they have any influence whatever in increasing the solubility of the urates in the fluids of the body. Again, the alkalies are supposed to facilitate the excretion of uric acid through the kidneys, but the evidence is "hopelessly contradictory," and Roberts denies that the most prolonged administration of them has any effect in warding off arthritic attacks. Indeed, the want of alkalinity in the blood, said to occur in gout, is by no means marked, and is equally present in many other forms of cachexia, in fevers, anæmia, and carcinoma. Are we left then without any remedies which can prevent the occurrence of the gouty paroxysm, the uratosis? At present we know of none which stop deposition when the blood is once fully charged. Since table-salt increases the rapidity with which precipitation occurs, we may indeed restrict its use to the smallest amount, but here again the wonderful power of the blood in maintaining a fixed percentage, and throwing off any excess, limits the results we might obtain. If the kidneys have an abnormally small appetite for urates, and are unable to seize and excrete the necessary amount, if they are so to speak too small for the body, and if no means exist for breaking up uric acid compounds into other substances, the only way to aid them is by limiting the nitrogenous intake, or theoretically at least by increasing fæcal excretion in place of renal. Thus we should reduce the albuminoid elements in the food, and as far as possible, without interfering with digestion, limit our patients to eggs, fish, and vegetable diet. Of course, when the condition of recurrent uratosis is well established, the amount of change we can effect may be very small, but in earlier and slighter cases very much can be done by the most careful regulation of the diet and management of the general health.

In the condition where uric acid gravel is the trouble complained of by the patient there is a very different state of things. There may be no tendency whatever to gout, the uric acid is not precipitated in the organism as a biurate, but in the urine as uric acid. And this precipitation too depends on the state of the urine. not on that of the blood. The quantity of salines, pigments, and uric acid present, together with the degree of acidity of the urine, are the chief factors. The last is, perhaps, the most important. We may, indeed, diminish slightly the amount of albuminous food as in gout, and we must increase rather than lessen the amount of salt consumed, but the all-important point is to insure that at no time in the twenty-four hours does the acidity of the urine reach a high figure.

In gravel the kidneys are throwing off more uric acid than the urine can retain in solution until voided. In gout they cannot eliminate enough to save the tissues from uratosis. During sleep and fasting the acidity of the urine reaches its maximum, and by the administration of citrate of potash and other alkalies between meals, and especially at bedtime, any premature deposit of uric acid in the kidneys or bladder may be generally controlled.

*(To be continued.)*

